# Recent advances in the knowledge of Mexican Alticinae (Coleoptera, Chrysomelidae)

**DOI:** 10.3897/zookeys.720.17790

**Published:** 2017-12-11

**Authors:** David G. Furth

**Affiliations:** 1 Department of Entomology, National Museum of Natural History, Smithsonian Institution, Washington, D.C. 20560, USA

**Keywords:** Mexico state records, Flea Beetles, indoor collecting, faunistics, new combinations, *Systena/ Prasona* and *Alagoasa*/*Kuschelina* confusion, *Alagoasa decemguttatus*, *Omophoita octomaculata*, Mexican biodiversity

## Abstract

The present study updates previously published biodiversity/faunistics of the flea beetles of Mexico published by the author after examination of 6132 specimens from 8 institutional collections. The following 9 genera were selected as indicators of the effects of known diversity only through examination of museum specimens (i.e., “indoor collecting”): *Alagoasa* Bechyné; *Asphaera* Chevrolat; *Capraita* Bechyné; *Disonycha* Chevrolat; *Kuschelina* Bechyné; *Omophoita* Chevrolat; *Prasona* Baly; *Systena* Chevrolat; and *Walterianella* Bechyné. From the specimens examined in these genera from the 8 collections, there were 394 new records for Mexican states of the 287 new species records representing 47% new records of the species recorded from those states. Total new state records 287 from 80 species. States with most new records: Chiapas (32); Nayarit (27); Sinaloa (24). 80 spp. (47%) with new state records. *Systena oberthuri* Baly is reported from Mexico for the first time. The current total of Alticinae in Mexico is 90 genera/626 species. The difficulties of the generic boundaries between *Systena* and *Prasona*, *Alagoasa* and *Kuschelina*; as well as the specific boundaries between *A. jacobiana* and *A. decemguttatus* and the specific level pattern variation in *Disonycha glabrata* and *Alagoasa decemguttatus* are discussed. *Kuschelina semipurpurea*, formerly placed in *Alagoasa*, is placed is considered as a new combination.

## Introduction

For about 30 years I have been studying the biodiversity/faunistics of the Central American Alticinae (Furth and Savini 1996, 1998, Furth et al. 2003), especially the Mexican Alticinae (Furth 2004, 2006, 2009, 2013). The current project is a continuation of research about the biodiversity/faunistics, distribution, and biogeography of the Alticinae (Flea Beetles) of Mexico. The author has conducted extensive field work in the majority of the 32 Mexican states as well as examination and determination of thousands of Mexican specimens from many museums in North America. Two previous published surveys by the author were of individual selected states. The first one from Chihuahua and Sonora (Furth 2009) revealed 26 genera with 70 species from Chihuahua, including 44 species new to Chihuahua, 9 species new to Sonora, 10 species new to Mexico, and two species new to science. The second from Oaxaca (Furth 2013) discovered 68 genera with 275 species (113 species known only from Oaxaca) in that state alone elevating the total recorded Alticinae fauna of Mexico to 90 genera and 625 species. This Oaxaca study demonstrated a very strong biogeographic affinity to the Neotropical Region. Some of the species in this Oaxaca study were at the time unidentifiable morpho-species and some either far from their known distribution or even new to science. The last comprehensive list of the entire Mexican Alticinae fauna was over 10 years ago and a lot of new specimen data has become accessible. There are still many more specimens in the remaining Alticinae genera currently being studied by the author from the institutions mentioned in the current study as well as others in a few other North American and Mexican institutions. Some details of the Mexican Alticinae fauna as well as about Mexican biogeography were included in Furth (2006) which recorded 89 genera and 524 species of Alticinae in Mexico.

Some of the author’s recent publications (mentioned above) concerning the Mexican fauna stressed the value of a combination of strategies for biodiversity research beginning with a comprehensive search of the literature examination, combined with fieldwork and extensive search of institutional collections. The current study concentrates on the latter of these strategies to uncover additional specimens and localities in order to better understand Mexican flea beetle diversity.

## Methods

During the past 10+ years the author has visited the institutions listed below and has pulled out all Alticinae from Mexico and borrowed them. For the current study nine genera, including the genera of the “Oedionychini/a” tribe/subtribe (*Alagoasa*, *Asphaera*, *Capraita*, *Kuschelina*, *Omophoita*, *Walterianella*), as well as a few other randomly selected genera (*Disonycha*, *Prasona*, *Systena*) were studied and determined to species. There were 6625 adult specimens studied and determined to species based on morphology and using literature and reference collections. The term species refers also to all taxa, including subspecies recorded from Mexico.

The institutional collections studied are: American Museum of Natural History (New York, New York, USA) [**AMNH**]; Monte L. Bean Museum of Brigham Young University (Provo, Utah, USA) [**BYU**]; Natural History Museum (London, United Kingdom) [**NHM**]; California Academy of Sciences (San Francisco, California, USA) [**CAS**]; California Department of Food and Agriculture, Sacramento, California, USA) [**CDFA**]; University of California Berkeley (Berkeley, California, USA) [**UCB**]; University of California Davis, Davis, California, USA) [**UCD**]; U.S. National Museum/NMNH (Washington, D.C., USA) [**NMNH**]; and a few specimens donated to the author by R. Wills Flowers (Florida State University, Tallahassee, Florida, USA) [**RWF**]. Specimens were studied and determined using a Leica MZ-APO dissecting stereomicroscope, as well as specimen reference collections at the U. S. National Museum of Natural History, Smithsonian Institution, Washington, D.C. USA [NMNH/NMNH], relevant literature in the author’s library and certain online references, e.g., MCZ Type Database.

The habitus photographic images were taken using the Visionary Digital BK Lab Imaging system outfitted with the Canon EOS 5D and a MP-E 65 mm 1–5× Canon macrolens. Stacked images were processed in part with Helicon Focus; final editing was done with Adobe PhotoShop. Specimens will be returned to their original institutional collections with some vouchers deposited at the NMNH.

Images of adult species for Figures 4–6, 8–9 were taken randomly from the Internet by searching for the species name. Figures 1–3 were taken by the author; Figure 7 was taken by K. Darrow.

In Table 2 (totaled in Table 1) there are some new state records from multiple institutional collections, but each new state record is only counted once (e.g., for *Alagoasa acutangula* Nayarit is a new state record and was recorded from AMNH, CAS, CDFA, and UCB, but it is only counted as a single state record). The taxa in Table 2 are referred to in the text as species, but a few are subspecies names.

The author follows a less popular classification, i. e., Alticinae rather than Alticini, as explained in several publications, e.g., Furth and Lee (2000), Furth and Suzuki (1998), Mohamedsaid and Furth (2011).

**Table 1.** Indoor Collecting. Numbers of specimens examined, by institutional collection. For Specimens Examined, the number in parentheses are those not determined to species.

Collection	Specimens Examined	New State Records/Collection
AMNH	374 (9)	30
NHM	120 (14)	1
BYU	309 (33)	29
CAS	1412 (93)	105
CDFA	374 (20)	27
UCB	3131 (223)	150
UCD	846 (97)	46
NMNH	59 (4)	6
TOTALS	6625 (493)	394/287*

*Repeats/Actual

**Table 2.** List of determined species by institution with state records (new state records in bold print).

Genus	Species	Author	Distribution	Amnh	Bmnh	Byu	Cas	Cdfa	Nhm	Ucb	Ucd	Rwf	Usnm	New Records	No. New Records
*Alagoasa*	*acutangula*	(Jacoby)	CHIS, COL, DGO, GRO, JAL, MEX, MOR, NL, OAX, VER	**NAY**		GRO, JAL,	CHIS, GRO, MOR, **NAY**, **SIN**, **SLP**	JAL, **MICH**, **NAY**	VER	CHIS, DGO, GRO, JAL, **MICH**, MOR, **NAY**, NL, **PUE**, **SIN**, VER	GRO, MOR, **PUE**, **SIN**		GRO, **QRO**	MICH, NAY, PUE, QRO, SIN, SLP	6
	*bipunctata*	(Chevrolat)	CHIS, DF, OAX, SLP, VER, YUC	CHIS, SLP, **QROO**, **TAMPS**, VER, YUC		**PUE**, SLP	VER		**MOR**, VER	CHIS, **QRO**, SLP, VER	SLP			MOR, PUE, QROO, QRO, TAMPS	5
	*chevrolati*	(Baly)	OAX, VER, YUC				VER		VER						
	*clypeata*	(Jacoby)	CHIS, DGO, HGO, MICH, OAX, TAB, VER			VER		CHIS, VER	VER	CHIS, **DGO**, OAX, VER	CHIS, VER	VER		DGO	1
	*donckieri*	(Jacoby)	GRO					**PUE**						PUE	1
	*extrema*	(Harold)	MOR, OAX, TAB, VER	**CHIS**, VER			**CHIS**		VER	VER	VER		**YUC**	CHIS, YUC	2
	*fimbriata*	(Forster)	GRO, MICH, MOR, OAX				MICH								
	*hoegei*	(Jacoby)	OAX, VER				VER								
	*inconspicua*	(Jacoby)	DGO, JAL	JAL		JAL	**SIN**			DGO, JAL, **SIN**				SIN	1
	jacobiana* [comb. n.]	(Horn)		CHIH, CHIS, DGO, JAL, MOR, NAY, OAX, SON, **SIN**, **YUC**		GRO, SON	CHIS, MOR, NAY, **SIN**, SON	COL, JAL, NAY, **SIN**	MEX	CHIH, CHIS, DGO, GRO, JAL, NAY, OAX, **SIN**, SON	CHIH, JAL, MOR, **SIN**			SIN, YUC	2
	*lateralis*	(Jacoby)	COL, GRO, JAL, MEX, MICH, MOR, NAY, OAX	NAY		**PUE**	**CHIH**, **COL**, MOR, NAY, **NL**, **SIN**, **VER**	**COL**, JAL, NAY, **SIN**	VER	GRO, MOR, NAY, **SIN**, **VER**	**CHIS**, MOR			CHIH, CHIS, COL, NL, SIN, VER	6
	*longicollis*	(Jacoby)	OAX				**VER**							VER	1
	semipurpurea**	(Jacoby)	VER	**CHIH**										CHIH	1
	*seriata*	(Baly)	GRO, MOR, OAX, PUE, VER	MOR			MOR, **SLP**, **TAMPS**, VER			**CHIS**, GRO				CHIS, SLP, TAMPS	3
	*tehuacana*	Bechyné	JAL, PUE					JAL		JAL					
	*tridecimmaculata*	(Jacoby)	GRO, MICH	**SIN**					**JAL**, **MEX**		**MOR**		GRO, MEX, MOR	JAL, MEX, MOR, SIN	4
	*trifasciata escuintla*	Bechyné	Mexico	**NAY**, **SLP**, **TAB**, **TAMPS**, **VER**		SLP, **VER**	**CHIS**, **MEX**, **NAY**, **TAMPS**, **VER**	CHIS, **JAL**, **NAY**, **SIN**	**VER**	CHIS, JAL, **NAY**, SLP, **TAB**, **VER**	**NL**, **TAMPS**, **VER**			CHIS, JAL, MEX, NAY, NL, SIN, SLP, TAB, TAMPS, VER	10
	*virgata*	(Harold)	CHIH, CHIS, COL, DGO, GRO, JAL, MEX, MOR, NAY, PUE, OAX, SIN, SLP, TAB, VER	GRO		JAL, NAY, PUE	CHIS, JAL, MOR, NAY, SIN, **SON**, VER	JAL, NAY, SIN	MEX, MOR, VER	CHIS, GRO, JAL, MOR, NAY, ?**QRO**, SIN, **SON**, VER, ?**YUC**	CHIH, CHIS, MOR, OAX, VER			?QRO, SON, ?YUC	3
*Asphaera*	*abdominalis*	(Chevrolat)	AGS, CHIH, CHIS, COAH, COL, DF, DGO, GRO, GTO, HGO, JAL, MEX, MICH, MOR, NL, OAX, SIN, SLP, TAMPS, VER, ZAC	HGO, SIN			CHIS, DGO, GTO, JAL, MICH, MOR, **NAY**, NL, OAX, SIN, SLP, **TAB**, VER, ZAC	JAL, MEX, MICH, **PUE**, SIN, SLP	DF, JAL, MEX, MOR	CHIH, CHIS, DGO, GRO, GTO, HGO, JAL, MEX, MICH, NL, OAX, PUE, **QRO**, SIN, TAMPs, VER, ZAC	AGS, HGO, JAL, MEX, MICH, **SON**, SIN, ZAC		**QRO**	NAY, PUE, QRO, SON, TAB	5
	*cyanopsis*	Harold	DF, DGO, OAX, SLP, TAB, VER	OAX, SLP		**PUE**, SLP	VER			**CHIS**, **PUE**, SLP, TAB, VER	VER			CHIS, PUE	2
	*lustrans*	(Crotch)	BC						**MEX**					MEX	1
	*mexicana*	(Harold)	CHIS, DGO, GRO, MICH, MOR, NAY, OAX, VER												
	*reichei*	(Harold)	CHIS, DF, OAX, SLP, VER				CHIS, **JAL**, **MOR**, **NAY**, VER		**MEX**	CHIS, **GRO**, **JAL**, **MICH**, **NAY**, **NL**, OAX, VER	VER			GRO, JAL, MEX, MICH, MOR, NAY, NL,	7
*Capraita*	*conspurcata*	(Jacoby)	CHIS, DF, DGO, GRO, GTO, HGO, MEX, MICH, MOR, OAX, PUE, VER	**SIN**			CHIS, DGO, MEX, PUE, **TAB**, VER			DF, DGO, MEX, MICH, PUE, **SIN**, VER	**SIN**			SIN, TAB	2
	*maculata*	(Harold)	CHIS, GRO, JAL, MEX, MOR, OAX, VER, YUC				JAL, **MICH**, **NAY**, **SIN**	GRO, JAL		GRO, **NAY**, **SIN**				MICH, NAY, SIN	3
*Disonycha*	*angulata*	Jacoby	SLP, TAB, VER, YUC			VER				**CHIS**, VER				CHIS	1
	*annulata*	Blake	Mexico					**JAL**						JAL	1
	*antennata*	Jacoby	COL, DGO, GRO, JAL, MEX, MICH, MOR, OAX, VER		MEX		MICH, MOR, **NAY**, **SIN**, VER	SIN		**CHIS**, GRO, JAL, MICH, **NAY**, OAX, **SIN**, VER	**CHIH**, MICH, **SIN**, VER			CHIH, CHIS, NAY, SIN	4
	*arizonae*	Casey	CHIH, DGO?, GRO, MOR?							CHIH, **COAH**, **NL**, **SON**	CHIH, **NL**			COAH, NL, SON	3
	*barberi*	Blake	GRO, SIN, VER				SIN	**MICH**		**NAY**, VER	SIN			MICH, NAY	2
	*brevilineata*	Jacoby	DGO, GRO, JAL, MOR, OAX		**MEX**		**CHIS**, MOR	JAL		**CHIS**, JAL, **NAY**				CHIS, MEX, NAY	3
	*brunneofasciata*	Jacoby	GRO, PUE, SLP							**OAX**, **SIN**				OAX, SIN	2
	*collata*	(Fabricius)	CHIH, COAH, DF, DGO, GTO, JAL, MEX, MICH, MOR, OAX, PUE, TAB, VER, YUC				DGO, JAL, MEX, MOR, **NAY**, PUE, **SIN**, **SON**	**SIN**, VER		DF, JAL, **NAY**, **SIN**	**NAY**, **SIN**			NAY, SIN, SON	3
	*dorsata*	Harold	MOR, OAX, TAB, VER, YUC			VER	**CHIS**, **JAL**, **MOR**, **NAY**, VER	**JAL**		**CHIS**, **NAY**, OAX, **SLP**, VER				CHIS, JAL, MOR, NAY, SLP	5
	*figurata*	Jacoby	AGS, CHIH, CHIS, COAH, COL, DF, DGO, GRO, GTO, JAL, MEX, MICH, MOR, NAY, OAX, SIN, TAB, VER, YUC	CHIH	MEX	CHIH, JAL, **SON**	CHIH, MOR, NAY, SIN,	JAL, MOR, SIN		CHIH, CHIS, COL, DF, DGO, GRO, GTO, JAL, MICH, NAY, OAX, SIN, **SON**, **TAMPS**, VER	CHIH, CHIS, JAL, MICH, NAY, SIN, **ZAC**			SON, TAMPS, ZAC	3
	*fumata fumata*	LeConte	BC, CHIH, CHIS, DGO, GRO, HGO, JAL, MEX, MICH, MOR, NL, OAX, PUE, SLP, SON, TAB, VER, ZAC	**COL**, TAB	MEX	JAL, MEX	CHIS, GRO, **NAY**, PUE, **SIN**, SON, ZAC	JAL, MOR, **SIN**		**BCS**, CHIS, **COL**, DGO, GRO, JAL, MICH, **NAY**, NL, PUE, **SIN**, SON, VER	CHIH, JAL			BCS, COL, NAY, SIN	4
	*glabrata*	(Fabricius)	BC, BCS, CAMP, CHIS, COL, DGO, GRO, JAL, MOR, NAY, OAX, PUE, SIN, SON, TAB, TAMPS, YUC, VER	COL, TAB	JAL, MOR	SON	BCS, **CHIH**, CHIS, JAL, **MEX**, MOR, NAY, **NL**, SIN, SON, TAMPS, VER	COL, JAL, NAY, SIN, SON		CAMP, **CHIH**, CHIS, COL, GRO, **GTO**, JAL, **MICH**, MOR, NAY, NL, OAX, **QROO**, SIN, SON, TAB, VER, YUC	GRO, JAL, MOR, NAY, SIN, **SLP**, SON, VER			CHIH, GTO, MEX, MICH, NL, QROO, SLP	7
	*guatemalensis*	Jacoby	CHIS, GRO, MOR, OAX, VER?				CHIS			CHIS, GRO, **JAL**, **TAB**, VER	MOR, **TAMPS**			JAL, TAB, TAMPS	3
	*jalapensis*	Blake	VER				**CHIS**			**CHIS**, **COL**, **SIN**	VER			CHIS, COL, SIN	3
	*knabi*	Blake	GRO							**OAX**, **VER**				OAX, VER	2
	*leptolineata texana*	Schaeffer	DGO, GRO, JAL, MOR, NL, OAX, QROO, TAMPS, YUC		**MEX**		**NAY**, **SON**			**CHIH**, **CHIS**, **COL**, **MICH**, **OAX**, **VER**	**CHIS**, MOR			CHIH, CHIS, COL, MEX, MICH, NAY, OAX, SON, VER	9
	*limbata*	Jacoby	DGO, MEX, MOR							DGO					
	*maculipes*	Jacoby	CHIS, VER	**SON**			**BCS**, **DGO**, **SON**	**COAH**		CHIS, **TAMPS**				BCS, COAH, DGO, SON, TAMPS	5
	*melanocephala*	Jacoby	VER				**CHIS**			**CHIS**, VER				CHIS	1
	*mexicana*	Jacoby	TAB, SLP, VER, YUC			**TAMPS**	VER			**BC**, **CHIS**, **DGO**, **NAY**, **OAX**, **PUE**, **SON**, TAB, VER				BC, CHIS, DGO, NAY, OAX, PUE, SON, TAMPS	8
	*militaris*	Jacoby	TAB, VER, YUC			**CHIS**	**CHIS**			**CHIS**				CHIS	1
	*nigrita*	Jacoby	OAX (Furth, 2013)					**COL**, **NAY**		**NAY**				COL, NAY	2
	*pluriligata*	LeConte	BC, CHIH, DGO, JAL, NAY, SIN, SLP, SON, VER		JAL	CHIH, JAL, SLP, SON	**CHIS**, JAL, NAY, SLP, SON, **TAMPS**, VER			**CHIS**, DGO, **OAX**, VER	**CAMP**, CHIH, DGO, **MEX**, **MICH**, SIN, SON			CAMP, CHIS, MEX, MICH, OAX, TAMPS	6
	*politula*	Horn	AGS, CAMP, CHIH, DF, DGO, GRO, GTO, HGO, JAL, MEX, MOR, OAX, PUE, QRO, SLP, SON, TAMPS, VER, ZAC	VER	DGO	CHIH	AGS, **CHIS**, DGO, JAL, **NAY**, SON			AGS, CHIH, DGO, GRO,GTO, HGO, JAL, **MICH**, **NAY**, **NL**, PUE, SLP, SON, ZAC	CHIH, DGO, HGO, JAL, PUE, ZAC,			CHIS, MICH, NAY, NL	4
	*procera*	Casey	NAY	NAY			NAY								
*Disonycha*	*quinquelineata*	(Latreille)	CHIS, COL, GRO, OAX, QROO, TAB, TAMPS, VER					**JAL**		VER				JAL	1
	*recticollis*	(Jacoby)	NAY, VER								VER				
	*sallei*	(Baly)	VER				VER								
	*scriptipennis*	(Jacoby)	CHIS, COL, DGO, GRO, MOR, NAY, OAX, YUC				CHIS, NAY			CHIS, **JAL**, **MICH**, **SIN**	GRO, JAL, NAY			JAL, MICH, SIN	3
	*subaenea*	Jacoby	DGO, GRO, MOR, OAX							**JAL**				JAL	1
	*tenuicornis*	Horn	CHIH, DGO, HGO				**CHIS**, **SIN**	HGO, **JAL**, **SIN**		CHIH, DGO, HGO, **NL**, **SIN**				CHIS, JAL, NL, SIN	4
	*trifasciata*	Jacoby	CHIS				CHIS			CHIS					
*Kuschelina*	*laeta*	(Perbosc)	TAMPS, VER	**COL**, **NAY**		**CHIH**	**BC**, **MOR**, **NAY**, **NL**, VER			**BCS**, **DGO**, **GTO**, **JAL**, **NAY**, **SIN**, **SON**				BC, BCS, CHIH, COL, DGO, GTO, JAL, MOR, NAY, NL, SIN, SON	12
	*modesta*	(Jacoby)	CHIH, CHIS, DF, DGO, GRO, GTO, HGO, MEX, MOR, OAX, PUE, SLP, TLAX, VER			CHIH	PUE			MEX	CHIH				
	*semipurpurea* ***	(Jacoby)		**CHIH**										CHIH	1
*Omophoita*	*aequinoctialis aequinoctialis*	(Linnaeus)	Mexico						**TAB**	**TAB**, **VER**				TAB, VER	2
	*affinis* ?	(Jacoby)	Mexico ?							**CHIS**				CHIS	1
	*cinctipennis*	(Chevrolat)	JAL, OAX, PUE, SLP, VER	SLP, VER			**NL**, SLP			PUE, SLP, VER	VER			NL	1
	*cyanipennis octomaculata* **** Crotch (= some aequinoctialis aequinoctialis & * punctulata* Bechyne & Bechyne)	(Crotch)	OAX, TAB, TAMPS, VER	**CHIS**, **NAY**, **SLP**, TAB, TAMPS, VER, **YUC**		**NL**, **SLP**, TAMPS	CHIS, **MEX**, **NL**, **SLP**, VER	VER	**CHIS**, TAB, VER	**CAMP**, CHIS, **COAH**, OAX, **SLP**, VER	**SLP**, TAMPS, VER			CAMP, CHIS, COAH, MEX, NAY, NL, SLP, YUC	8
	*quadrinotata centraliamericana*	Bechyné	OAX, TAB, VER	TAB, VER			**CHIS**, **MEX**, VER		TAB, VER	**CHIS**, TAB, VER	VER	VER		CHIS, MEX	2
	*recticollis*	(Baly)	CHIS, HGO, OAX, TAB, TAMPS, VER				VER			CHIS, VER					
	*violacea*	Jacoby	GRO	**NAY**			**NAY**			**CHIS**, **JAL**, **MICH**, **NAY**, **SON**	**SIN**			CHIS, JAL, MICH, NAY, SIN, SON	6
*Prasona*	*viridis*	Baly	VER			**PUE**	VER	**JAL**?		**PUE**, VER				JAL?, PUE	2
*Systena*	*abbreviata*	Jacoby	PUE			**MOR**	PUE, **VER**			PUE				MOR, VER	2
	*bitaeniata*	LeConte	CHIH			CHIH	**BC**				**VER**			BC, VER	2
	*blanda*	Melsheimer	BC?, CHIH, JAL, MICH, NL, SIN, SLP?, SON, TAB, VER			CHIH, JAL	**NAY**, NL, **PUE**, **SLP**, SON			**CHIS**, JAL, **MOR**, **NAY**, SIN, **SLP**	CHIH, **COAH**, JAL, SIN, SON			CHIS, COAH, MOR, NAY, PUE, SLP	6
	*contigua*	Jacoby	CHIS, GRO, GTO, HGO, OAX, SON?, TAMPS, VER?, ZAC			CHIS, HGO, **SLP**	CHIS, **VER**	**JAL**		**NAY**, **NL**, **PUE**, **QRO**, **SIN**, **SON**, **VER**				JAL, NAY, NL, PUE, QRO, SIN, SLP, SON, VER	9
	*discicollis*	Clark	CAMP, CHIH, DF, DGO, GTO, JAL, MEX, MICH, TAB, TAMPS, VER?, ZAC	MICH		DGO	DF, JAL			DGO, **HGO**, MEX, MICH	JAL, MICH, ZAC			HGO	1
	*gracilenta*	Blake	NL	**CHIH**		**CHIH**, **CHIS**, **SLP**, **SON**, **TAMPS**	**MOR**, SLP, TAMPS, **VER**, **ZAC**	**CHIS**		**CHIH**, **GRO**, **PUE**, **SIN**, **SLP**, **VER**	**CHIH**, **HGO**, **SLP**, **VER**		**QROO**, **ZAC**	CHIH, CHIS, GRO, HGO, MOR, PUE, QROO, SIN, SLP, SON, TAMPS, VER, ZAC	13
	*laevis*	Blake					**BC**							BC	1
	*marginata*	Jacoby	PUE, VER				**CHIS**							CHIS	1
	*nigroplagiata*	Jacoby	AGS, CHIH, DF, DGO, GTO, GRO, JAL, MICH, MOR, OAX, PUE, VER			CHIH, **CHIS**, GRO, JAL, MOR, OAX, **SLP**, **SON**, VER	MOR, PUE, **SIN**, **TAMPS**, VER			DGO, GRO, GTO, JAL, MICH, MOR, PUE, **SIN**	CHIH, JAL, MOR, **SIN**			CHIS, SIN, SLP, SON, TAMPS	5
	*oberthuri*	Baly					**CHIS**							CHIS	1
	*pectoralis*	Clark	CHIS, GTO, OAX, VER				VER			CHIS, **PUE**, VER	**GRO**			GRO, PUE	2
	*s-littera*	(Linnaeus)	CHIS, GTO, TAB, VER			VER	VER	VER		CHIS, TAB, VER	VER				
	*salvini*	Jacoby	CHIS				CHIS				CHIS				
	*semivittata*	Jacoby	BCS, GRO, GTO, HGO, MEX, MOR, NL, OAX, SIN	**CHIH**		**CHIH**, **SON**	**PUE**	MOR		**CHIH**, **CHIS**, **NAY**, **SON**	**CHIH**, **CHIS**, **JAL**, **PUE**, SIN, **SON**, **ZAC**			CHIH, CHIS, JAL, NAY, PUE, SON, ZAC	7
	*subcostata*	Jacoby	MICH, MOR, VER				**GRO**	MICH		**GRO**, **JAL**, **NAY**, VER	MICH			GRO, JAL, NAY	3
	*subrugosa*	Jacoby	GTO, MICH, MOR				**TAB**	**GRO**		**GRO**, **PUE**	**ZAC**			GRO, PUE, TAB, ZAC	4
	*sulphurea*	Jacoby	CHIH, DGO, GRO, MOR, OAX							CHIH, **CHIS**, **PUE**, **SON**	GRO, MOR, **PUE**			CHIS, PUE, SON	3
*Systena*	*thoracica*	Jacoby	CAMP, HGO, PUE, QROO, TAB, VER	**CHIS**, **MEX**, VER			**JAL**, **NAY**, **SLP**, VER			**CHIS**, **JAL**, **NAY**, **SIN**, TAB, VER	JAL, VER			CHIS, JAL, MEX, NAY, SIN, SLP	6
	*undulata*	Jacoby	AGS?, CAMP?, GRO?, GTO, MOR?, VER			**MOR**	**MEX**			**GRO**, **MICH**				GRO, MEX, MICH, MOR	4
	*variabilis*	Jacoby	CHIH, CHIS, COL, DGO, GRO, GTO, MICH, MOR, NAY, OAX, VER			GRO, MOR, **PUE**, **SLP**, **TAMPS**, VER	CHIH, **JAL**, MOR, NAY, **SIN**, VER	**JAL**, **SIN**		CHIH, **JAL**, MICH, MOR, NAY, **QRO**, **SIN**, VER	CHIH, MICH, **SIN**, VER			JAL, PUE, QRO, SIN, SLP, TAMPS	6
*Walterianella*	*biarcuata*	(Chevrolat)	CHIS, VER				**NAY**, VER		VER	VER				NAY	1
	*durangoënsis*	(Jacoby)	CHIH, DGO			**SON**	**NAY**, **SON**			DGO, **JAL**	CHIH, **SIN**		DGO	JAL, NAY, SIN, SON	4
	*inscripta*	(Jacoby)	OAX, SLP, VER			SLP	**CHIS**	VER		**CHIS**, VER				CHIS	1
	*oculata* ?	(Fabricius)	VER				VER			VER					
	*signata*	(Jacoby)	CHIS, JAL, TAB, TAMPS, VER, YUC	**QROO**, VER, YUC			CHIS, **MOR** ?,VER		VER	CHIS	**SLP**?			MOR?, QROO, SLP?	2
	*sublineata*	(Jacoby)	OAX, TAB, VER, YUC	**QROO**, VER			**CHIS**, VER			**CHIS**, **SLP**, VER				CHIS, QROO, SLP	3
	*tenuicincta*	(Jacoby)	SLP, TAB, VER			SLP	**CHIS**, **NAY**, SLP, VER							CHIS, NAY	2
	*venustula*	(Schaufuss)	CHIS, COL?, GRO, JAL, MICH, MOR, NAY?, QROO, TAMPS?, VER, YUC	VER, YUC		**NAY**, **SLP**	**COL**, JAL, MOR, **NAY**, **OAX**, SLP, **SON**	JAL, MICH, **NAY**	JAL	JAL, **SON**	MOR, NAY, **SIN**, VER	CHIS		COL, NAY, OAX, SIN, SLP, SON	6
**TOTAL**															**287**

* This species has apparently been confused with *A. 10-guttatus* (Fabricius); therefore, most *A. 10-guttatus* records are actually *A. jacobiana* (originally described and only known from USA: AZ, TX). ** *Alagoasa semipurpurea* = comb. n., this species should be placed in *Kuschelina*. *** *Kuschelina semipurpurea* = comb. n., see *Alagoasa*. **** *aequinoctialis aequinoctialis* (= *cyanipennis octomaculata* Crotch & *punctulata* Bechyne & Bechyne). BUT, re Blake 1931 *O. aequinoctialis (s.s.)* has a black metasternum & black metafemora. Indeed there seems to be some differences consistent with this & an elytral pattern different where *aequinoctialis (s.s.)* has the median/cental spots more rounded & only slightly angled whereas for *O. cyanipennis* 8-maculata they are more distincly angled and slender.

## Results

**Map 1.**
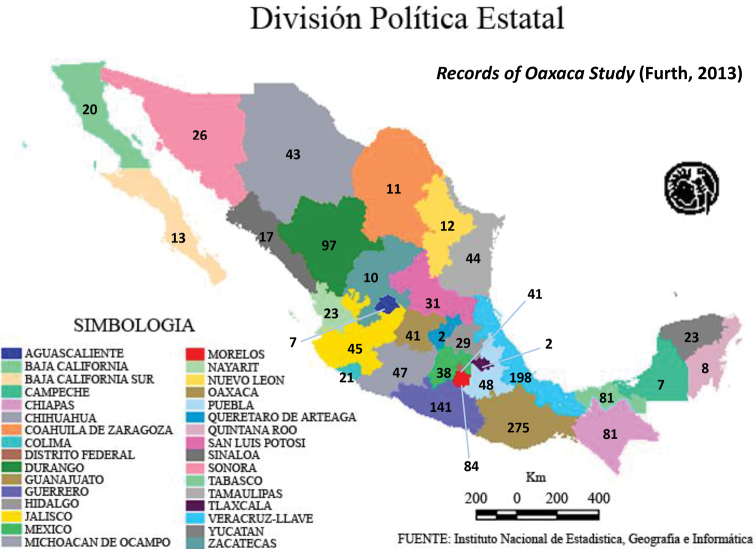
Species numbers by states from Furth (2013).

**Map 2.**
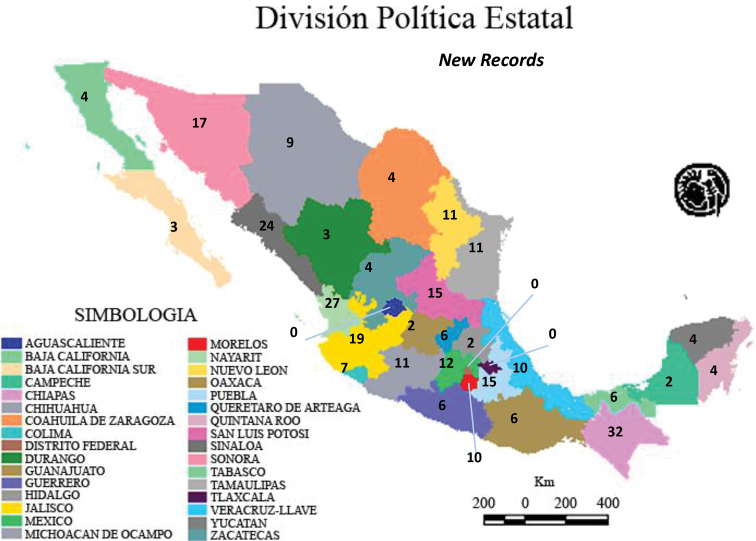
New species records by states from the current study.

**Map 3.**
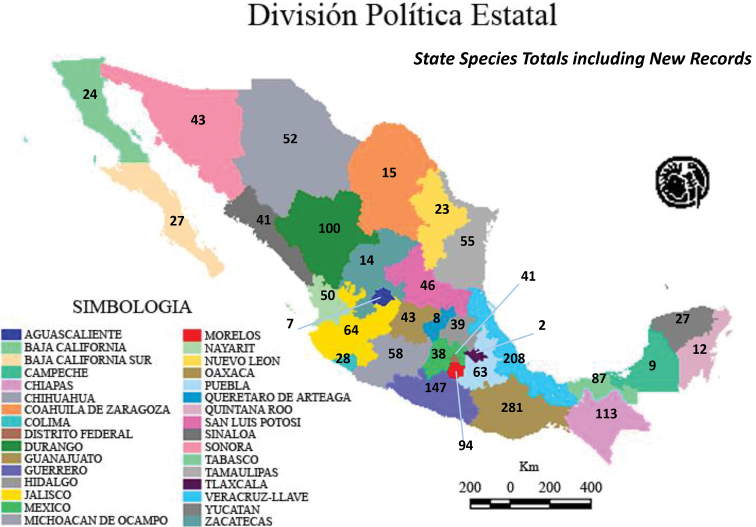
Total species records by states as of the current study.

Although 6625 Mexican Alticinae specimens from nine institutional collections were studied (see list in the Methods section above) some of these (493) that could not be determined reliably to recorded species (Table 1); therefore, a total of 6132 specimens were determined to species. Table 1 also demonstrates that a total of 394 new state records were found in these 8 collections, but 107 were repeated so that actually there are 287 new state records. The specimens studied belonged to a somewhat random assortment of genera, including the subtribe Oedionychina (*Alagoasa*, *Asphaera*, *Capraita*, *Kuschelina*, *Omophoita*, *Walterianella*) and *Disonycha*, *Prasona*, *Systena*. The genera with the most specimens belonged to *Alagoasa*, *Asphaera*, *Omophoita*, *Disonycha*, and *Systena*. Table 2 lists only the species in these genera with the new state records as discovered in the current study with those new state records in bold type; the full distribution of each species can be determined by combining these with the distributions in Furth (2006, 2009, 2013). Map 1 demonstrates the number of species by state as of Furth (2013) in comparison to the current study illustrating in Map 2 only the new species records by states and in Map 3 the total species per state.

**Figure 1.**
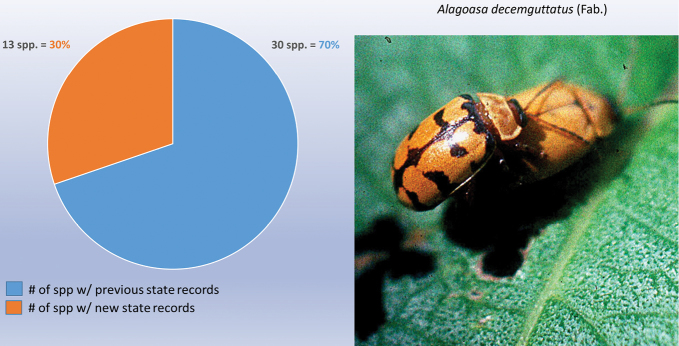
*Alagoasa* Bechyné new state records versus previously recorded state records.

**Figure 2.**
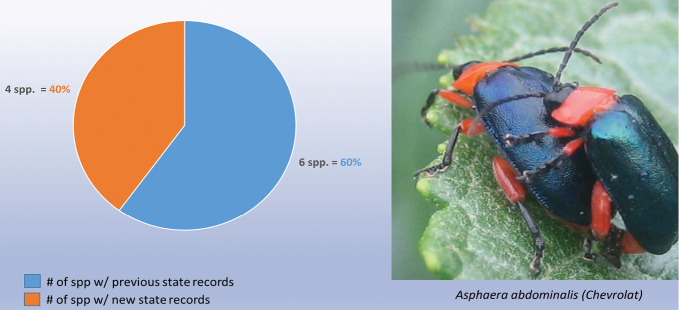
*Asphaera* Chevrolat new state records versus previously recorded state records.

**Figure 3.**
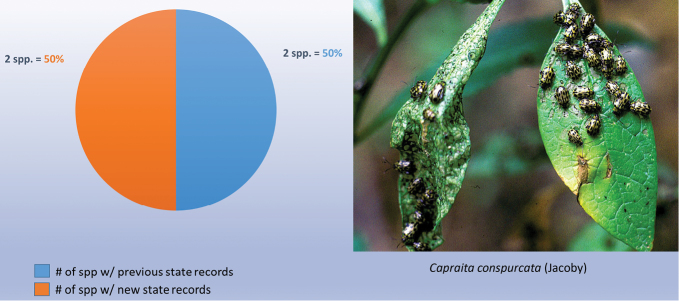
*Capraita* Bechyné new state records versus previously recorded state records.

**Figure 4.**
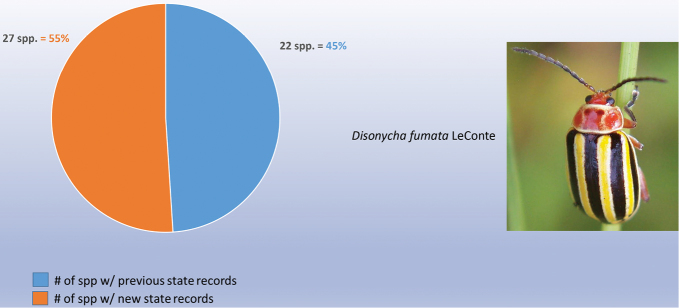
*Disonycha* Chevrolat new state records versus previously recorded state records.

**Figure 5.**
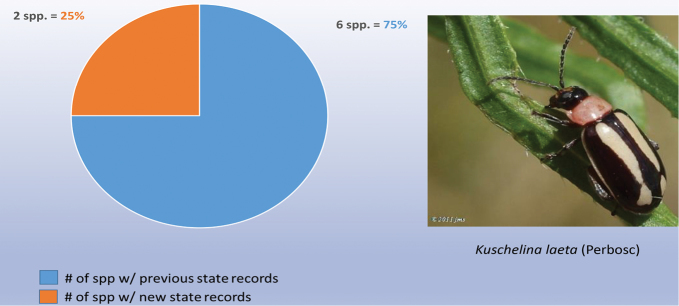
*Kuschelina* Bechyné new state records versus previously recorded state records.

**Figure 6.**
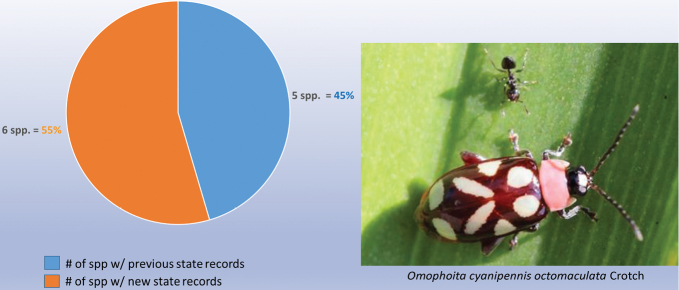
*Omophoita* Chevrolat new state records versus previously recorded state records.

**Figure 7.**
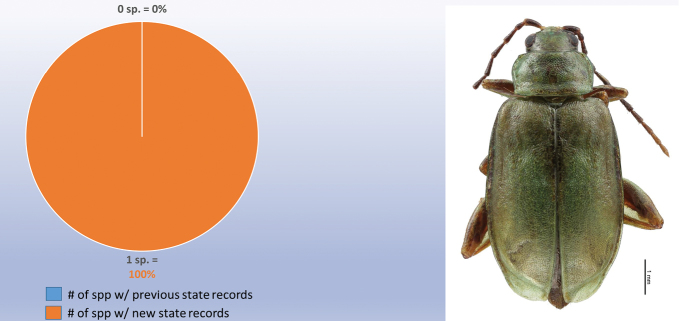
*Prasona* Baly new state records versus previously recorded state records.

**Figure 8.**
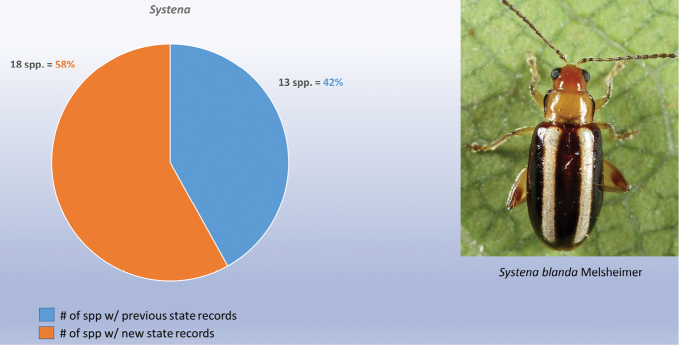
*Systena* Chevrolat new state records versus previously recorded state records.

**Figure 9.**
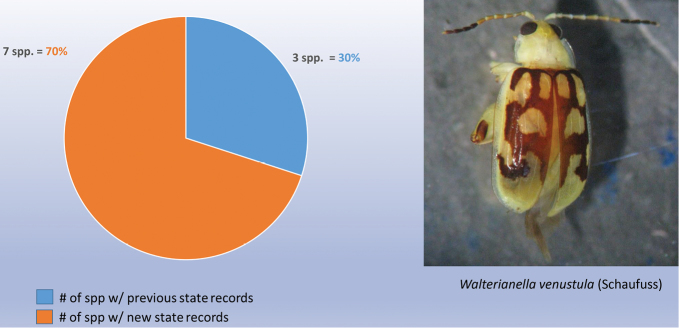
*Walterianella* Bechyné new state records versus previously recorded state records.

**Figure 10.**
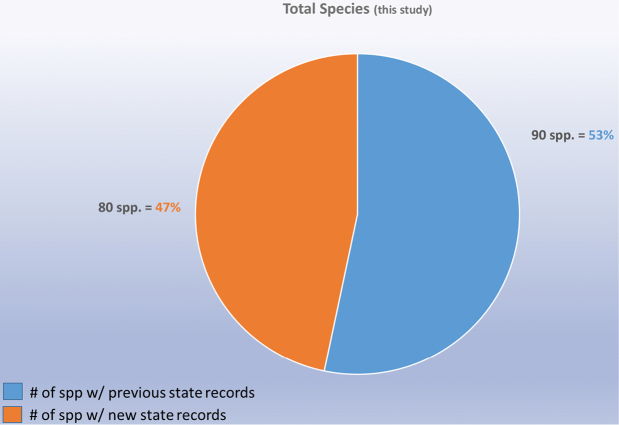
Summary of all new records for selected genera.

As indicated in Table 2 the nine genera studied have a high percentage of new state records based only on examination of these institutional collections, as follows: *Alagoasa* (13 of 18 species with new state records of the 44 species recorded from Mexico) [see also Fig. 1]. However, the two apparent new state records (SIN and YUC) for *A. jacobiana* are not included as new records because of its confusion with *A. decemguttata*; *Asphaera* (4 of 4 species of the 10 species recorded from Mexico) [see also Fig. 2]; *Capraita* (2 of 2 species of the 4 species recorded from Mexico) [see also Fig. 3]; *Disonycha* (27 of 33 species of the 49 species recorded from Mexico) [see also Fig. 4]; *Kuschelina* (2 of 3 species of the 8 species recorded from Mexico) [see also Fig. 5]; *Omophoita* (6 of 7 species of the 13 species record from Mexico) [see also Fig. 6]; *Prasona* (1of 1 species of the 1 species recorded from Mexico) [see also Fig. 7]; *Systena* (18 of 20 species of the 31 species recorded from Mexico) [see also Fig. 8]; and *Walterianella* (7 of 8 species of the 10 species recorded from Mexico) [see also Fig. 9]. Thus, there are 80 species of the 97, or almost 83% of the species examined from the nine institutional collections with new state records, and this is 47% of the total 170 species in these genera recorded from Mexico (Fig. 10).

Of special note in Table 2 are the confirmed species determinations that verify some questionable state records indicated in Furth (2006). They are as follows: *Disonycha guatemalensis* from Veracruz; *Omophoita affinis* from Mexico, a state record for a species only recorded previously as from the country of Mexico; *Systena bitaeniata* from Veracruz; *Systena blanda* from San Luis Potosi; *Systena contigua* from Sonora and Veracruz; *Systena undulata* from Guerrero and Morelos; *Walterianella venustula* from Colima.

*Systena oberthuri* Baly is reported for the first time from Mexico (Table 2; Fig. 12).

**Figure 11.**
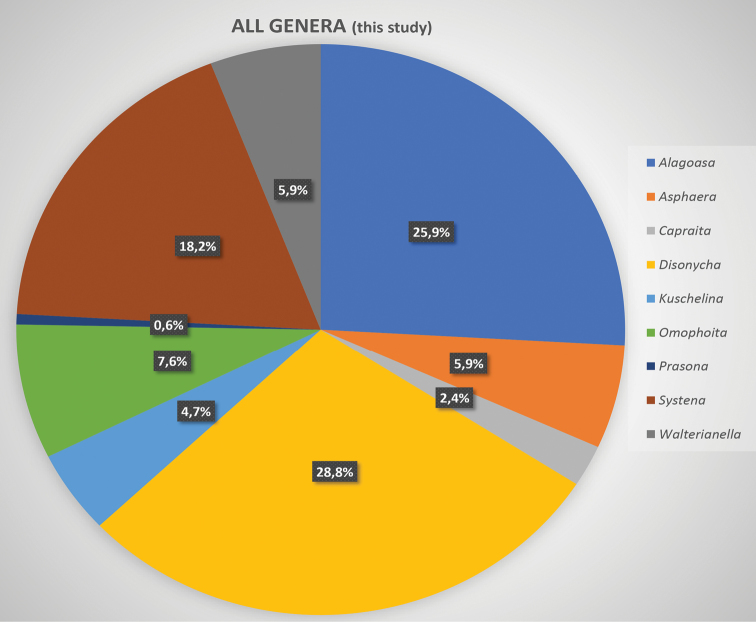
Summary of all genera with species percentages.

From the current study, it is evident that the numbers of recorded species have changed, in some cases significantly (see Maps 1, 2, 3). Map 2 shows these new records clearly (the details are in Table 2). The Mexican states with the most new species records are Chiapas (32), Nayarit (27), and Sinaloa (24).

## Taxonomic problems

### Generic level confusion

**Figure 12.**
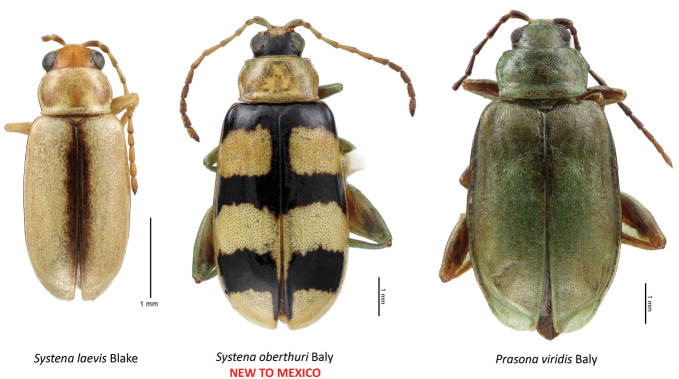
*Systena* and *Prasona* generic confusion.

*Systena* versus *Prasona* (Fig. 12). There has long been some confusion concerning the genus *Prasona* Baly as to whether it is synonymous with *Systena* Chevrolat. *Prasona viridis* Baly from Mexico is the type species (Baly 1861) and the only one recorded from Central America (Furth and Savini 1996) and eight other species from various parts of South America (Bechyné 1971). *Prasona* seems to have the primary characteristics of *Systena*, but seems only to differ in being much larger in size than species of *Systena*. *Prasona* was placed near *Systena* and *Cyrsylus* in the “catalog phylogeny” (Furth and Suzuki 1998) of Bechyné (1971).

*Alagoasa* versus *Kuschelina* (Fig. 17). *Kuschelina* Bechyné differs from *Alagoasa* Bechyné by having moniliform antennal segments; smaller eyes (diameter less than 3 times as small as width of frons); head (frons/vertex) rugosely punctured; male terminal sternite ventrally without depression; epipleura bent/slanted downward, i.e., visible in lateral view; elytral pleura narrow, not grooved or explanate; antero-lateral corners of pronotum only slightly protruding (not appearing to surround eyes/head; body shape elongate oval (not very rounded). The first placement of North American *Oedionychus* Berthold or *Oedionychis* Latreille (most *Alagoasa* were placed in one of these generic names previously) into *Kuschelina* was by Balsbaugh and Hays (1972) [for 14 spp.]. In Riley et al. (2003) there were 28 species of *Oedionychis* Latreille listed (following Wilcox 1975), but only one (*K. scripticollis* (Say) was noted as a new combination; however, many of the others are also technically new combinations, e.g., *K. amplivittata* (Blake), *K. barberi* (Blake), *K. flavocyanea* (Crotch), *K. jacobiana* (Horn), etc. Based on the above characters the author considers *K. semipurpurea* (Jacoby), formerly placed in *Alagoasa*, to be a new combination.

### Intra-specific variation:

**Figure 13.**
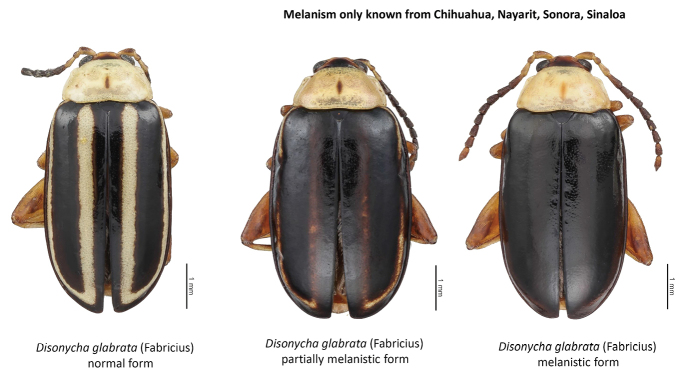
*Disonycha glabrata* (Fabricius) species color forms.

*Disonycha glabrata* (Fabricius) (Fig. 13). Because of melanization of the elytra there are intermediate and dark forms; known only from Chihuahua, Nayarit, Sonora, and Sinaloa.

**Figure 14.**
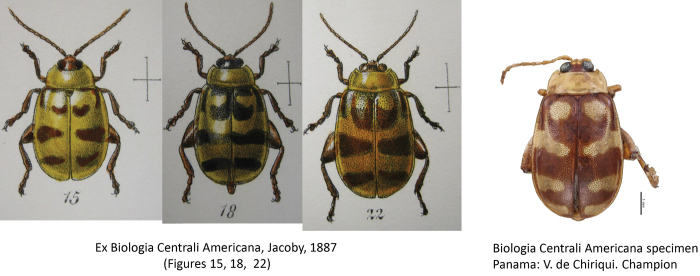
*Alagoasa decemguttata* (Fabricius) intraspecific variation.

**Figure 15.**
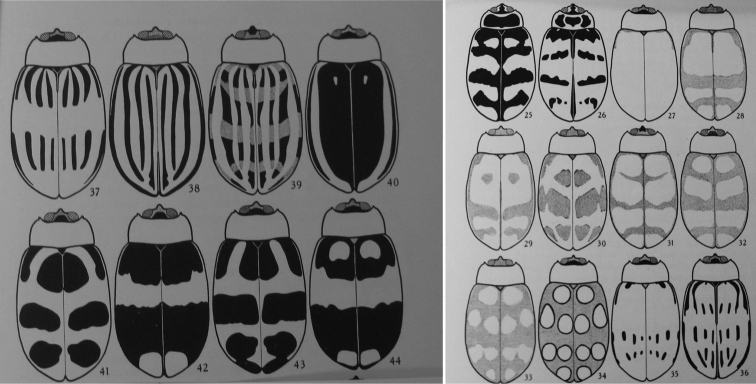
*Alagoasa decemguttata* intraspecific variation (ex Bechyné, 1955).

*Alagoasa decemguttatus* (Fabricius) (Figs 14, 15). Intra-specific variation in this species is well-documented in the literature (Jacoby 1886; Bechyné 1955).

### Species confusion (see Table 2):

**Figure 16.**
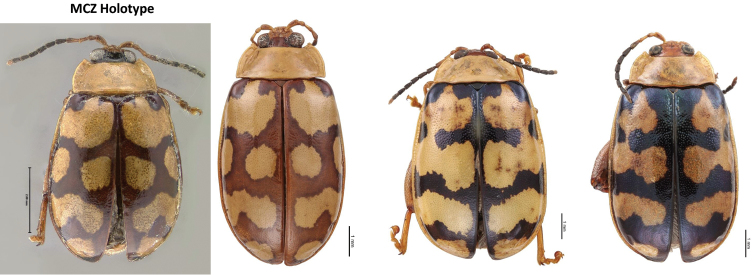
*Alagoasa jacobiana* (Horn) species confusion and intraspecific variation.

*Alagoasa decemguttatus* versus *A. jacobiana* (Horn) (Figs 14, 16). *Alagoasa decemguttatus*: Some confusion with this species. According to Bechyné (1971) *A. decemguttatus* is only from South America and, therefore, most *A. decemguttatus* recorded from Mexico are probably *A. jacobiana*; therefore, some records for *A. jacobiana* may be new records because of this confusion.

**Figure 17.**
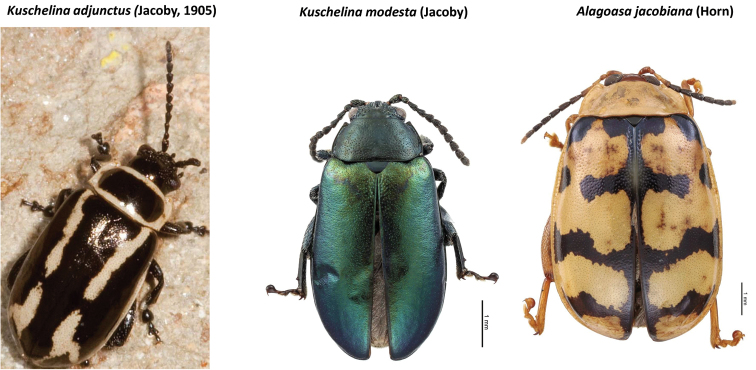
*Alagoasa* and *Kuschelina* generic confusion.

*Alagoasa duodecimmaculata* (Jacoby): There is some confusion of this species with *A. trifasciata escuintla* Bechyné & probably most specimens are *A. trifasciata escuintla*. Its occurrence in Mexico is still somewhat in question (Furth 2006).

*Omophoita cyanipennis octomaculata* Crotch or *O. octomaculata*: There has historically been confusion about the exact identity of *O. aequinoctialis aequinoctialis*. In this study, the author considers records of *O. a. aequinoctialis* to consist of both *O. cyanipennis octomaculata* Crotch and *O. punctulata* (Bechyné & Bechyné). However, according to Blake (1931) *O. aequinoctialis* (*s.s.*) has a black metasternum and black metafemora. Indeed, there seems to be some consistent differences between these and an elytral pattern different where *aequinoctialis* (*s.s.*) has the median/central spots more rounded and only slightly angled, whereas for *O. cyanipennis octomaculata* they are more distinctly angled and slender.

## Discussion

Interesting that this study did not reveal new genera and only discovered one new species for Mexico (Furth 2004, 2006, 2009, 2013). This may indicate that the number of described species found in Mexico may be reaching the level of being relatively well known. Only one new species record for Mexico (*Systena oberthuri*) was discovered in this study. However, based on the athor’s previous and current research, there are certainly many additional undescribed species living within Mexico; probably as many as another 300–400 species. Few other Central American countries have been surveyed for Chrysomelidae. Only Costa Rica is relatively well-known with 350 species in 89 genera (Furth et al. 2003) with only 113 species in 43 genera known previously from the literature. Panama is poorly-known and has 270 species in 70 genera recorded (Furth and Savini 1996, 1998).

Indoor Collecting (Table 1):

The author is not aware of any references in the literature referring to “indoor collecting”, especially in the meaning used in this study. One interview in 2015 of Dr. Art Evans referred to this term for picking up a beetle collection, something the author of this study has been doing for 50 years. However, the meaning for the current study refers to visiting institutional or private scientific collections for the purposes of scientific research, e.g., systematic revisions, faunisitcs, biodiversity, biogeographical, etc. Most biologists interested in nature, prefer the fun of “outdoor collecting” coupled with subsequent study of material and data from this back home, “indoor collecting” can be just as fun and usually even more productive scientifically; as evidenced from the current study. This kind of work is certainly nothing new, all students and professional biologists conduct this kind of work because the wealth of information “hiding” in such collections is phenomenal, vast, full of valuable information that can help answer many scientific questions and enhance most studies, and for the most part except data already published (at least in entomology), not available elsewhere. The current study is an example of “indoor collecting” where a large percentage (47%) of new Mexican state records were discovered.

Although the choice of the 9 genera and 8 collections for this study was rather random they still provide a very good example of the scientific value of collections. The author plans to continue such research on the Mexican Flea Beetle fauna based on much more specimen material he has borrowed from the same (and eventually other) collections. The 8 collections sampled represent a large percentage of the collections not yet studied by the author likely to have material from Mexico, notable exceptions are the collections of the Los Angeles County Museum, Texas A. & M. University, and, of course, the Universidad Nacional Autónoma de Mexico.

There is no particular pattern to the states with the most new records (Map 2). This is probably only an artifact of the historical collecting of individuals whose material is associated with the nine collections sampled. One of the 3 states with the most new records is Chiapas that is one of several southern states with strong tropical biogeographical affinities (Furth 2013). As evident in Map 2, this study produced new state records in all but three states (Aguascalientes, Distrito Federal, Tlaxcala) and there were new records with 10 or more in 13 states, i.e., over 30%; this is a testament to the value of “indoor collecting”. In previous studies of the Mexican Alticinae fauna some records were questionable because of unclear label data, unclear assignment in the literature, etc. (Table 2), but in the current study of the specimens in these 8 collections some of these were confirmed or enhanced for 7 species (see results above) for eight of the state, including one for the country.

In the Results section above the author has pointed out and attempted to clarify the taxonomic confusion at the generic and specific levels that came to light during the current study. There is no real need to elaborate in detail about these. Resolution of the confusion between *Systena* and *Prasona* requires considerably more study, but it is quite possible that *Prasona* will become a synonym of *Systena*. The confusion within the “Oedionychina” of *Alagoasa* and *Kuschelina* has caused some problems in faunistic studies in the Nearctic and Neotropical Regions, e. g., Riley et al. (2003). In the Results section above the author attempts to explain his interpretation of these two genera and to clarify the morphological differences, as well as to point out some new combinations created by this confusion.

Therefore, there are 80 species of the 96 species found in this study from 8 collections, or 83%, with new state records, and this is 47% of the total 170 species in these genera recorded from Mexico (Fig. 10). Figure 11 illustrates the nine genera in this study and their species percentages of the total.

In conclusion, the author hopes that not only does this study of a relatively few collections and genera significantly increase the knowledge of the Mexican Flea Beetle fauna, but also that it demonstrates the value of “indoor collecting” as an integral part of any biodiversity and faunistic research.
